# HECW1 induces NCOA4-regulated ferroptosis in glioma through the ubiquitination and degradation of ZNF350

**DOI:** 10.1038/s41419-023-06322-w

**Published:** 2023-12-04

**Authors:** Yuancai Lin, Hailong Gong, Jinliang Liu, Zhiwen Hu, Mingjun Gao, Wei Yu, Jing Liu

**Affiliations:** 1 Department of Neurosurgery, Shengjing Hospital of China Medical University, 36 Sanhao Road, 110000 Shenyang, China; 2 Liaoning Clinical Medical Research Center in Nervous System Disease, 7 Mulan Road, 117000 Benxi, China

**Keywords:** Cell death, Cancer genomics

## Abstract

Tumor suppression by inducing NCOA4-mediated ferroptosis has been shown to be feasible in a variety of tumors, including gliomas. However, the regulatory mechanism of ferroptosis induced by NCOA4 in glioma has not been studied deeply. HECW1 and ZNF350 are involved in the biological processes of many tumors, but their specific effects and mechanisms on glioma are still unclear. In this study, we found that HECW1 decreased the survival rate of glioma cells and enhanced iron accumulation, lipid peroxidation, whereas ZNF350 showed the opposite effect. Mechanistically, HECW1 directly regulated the ubiquitination and degradation of ZNF350, eliminated the transcriptional inhibition of NCOA4 by ZNF350, and ultimately activated NCOA4-mediated iron accumulation, lipid peroxidation, and ferroptosis. We demonstrate that HECW1 induces ferroptosis and highlight the value of HECW1 and ZNF350 in the prognostic evaluation of patients with glioma. We also elucidate the mechanisms underlying the HECW1/ZNF350/NCOA4 axis and its regulation of ferroptosis. Our findings enrich the understanding of ferroptosis and provide potential treatment options for glioma patients.

## Introduction

Gliomas are characterized by their highly invasive nature and high fatality rate [1, 2]. Despite substantial advances in surgical interventions and chemoradiotherapy, the median survival time of patients with glioma remains poor, prompting the need for novel strategies for the treatment of glioma in humans [3].

Cell death is key to maintaining the stability of cell populations; thus, research on regulated cell death has contributed important advances to tumor treatment [4, 5]. Ferroptosis, a new type of cell death, has become an increasingly popular topic of research since its discovery in 2012 [6]. Unlike other cell death mechanisms, the accumulation of iron and reactive oxygen species (ROS), reduction or disappearance of mitochondrial ridges are unique features of ferroptosis [7–9]. Nuclear receptor coactivator 4 (NCOA4) binds to ferritin heavy chain 1 and transports it to lysosomes for degradation, which enables it to induce ferroptosis in cells [10–12]. Growing evidence elucidates that ferroptosis has complex regulation on the biological process of tumor [5]. A variety of ferroptosis inducers, including both laboratory agents (erastin and RSL3) and approved drugs, exhibit promising tumor inhibition effects [5]. Therefore, the strategy of inducing ferroptosis to treat tumors has considerable research potential.

Ubiquitination describes the widespread post-translational modification of proteins in eukaryotes that regulates numerous biological processes [13, 14]. ATP, ubiquitin-activating enzyme(E1), ubiquitin-coupling enzyme(E2) and ubiquitin ligasethe(E3) are indispensable for the ubiquitination and degradation of proteins [15–17]. E3 determines the specificity of ubiquitin-substrate binding, which makes it a hot topic in the study of ubiquitination regulation [18–21]. E3 ubiquitin protein ligase 1 containing the HECT, C2, and WW domains (HECW1), belongs to the HECT family, was first identified in brain tumors [22, 23]. However, relatively little research has been conducted on HECW1, despite the fact that it enhances p53-mediated apoptosis in a manner independent of ubiquitin ligase activity [24]. Low expression of HECW1 is related to unfavorable prognosis, higher tumor staging, and stronger resistance to targeted drugs in patients with renal clear cell carcinoma [25]. In contrast, HECW1 promotes the progression of malignant behavior in non-small cell lung cancer by mediating the ubiquitination and degradation of Smad4 [26]. However, the study of HECW1 in glioma has not been reported.

Zinc finger protein 350 (ZNF350) is a typical KRAB-containing zinc finger protein identified in a yeast two-hybrid analysis of BRCA1 (breast cancer type 1 susceptibility protein)-related proteins [27]. ZNF350 has been identified as a transcriptional suppressor that can either form complexes with other proteins or play a direct transcriptional suppressive role in a single-factor form [28–32]. ZNF350 therefore plays an important role in the development of various tumors [31, 33, 34]. However, the potential effect and mechanism of ZNF350 in glioma have not yet been clarified.

In the study, we found that HECW1 blocked the progression of glioma cells and enhances iron accumulation and lipid peroxidation (LPO), which is caused by inducing NCOA4-mediated ferroptosis. Through screening and verification, we confirmed that ZNF350 mediated the positive regulation of HECW1 on NCOA4. In detail, The protein stability of ZNF350 was disrupted by HECW1-induced ubiquitination. ZNF350 inhibits the transcriptional activity of NCOA4 by directly binding to the transcriptional initiation site. Here, we demonstrated the regulatory mechanism of the HECW1/ZNF350/NCOA4 pathway on ferroptosis in gliomas, which might provide a novel strategy for glioma treatment.

## Materials and methods

### Data sources

Expression data and the clinical data of corresponding patients were downloaded from the Chinese Glioma Genome Atlas (CGGA) and The Cancer Genome Atlas Research Network (TCGA) databases [35, 36]. Gene expression boxplots were downloaded from the gene expression profiling interactive analysis (GEPIA) [37]. Transcription factors were predicted using the HumanTFDB database [38] and ubiquitination substrates were predicted using the UbiBrowser database [39].

### Cell culture

The cell lines were purchased from the Chinese Academy of Sciences (Shanghai, China) and ScienCell Research Laboratories (Carlsbad, CA, USA). Glioma cells (U87MG-Cat No.TcHu138, U251-Cat No.TcHu58) were cultured in Dulbecco’s modified Eagle’s medium (HyClone, UT, USA), while Normal human astrocytes (HA-Cat No. 1800) were cultured in astrocyte medium (ScienCell). The medium was supplemented with 10% fetal bovine serum (Gibco, NY, USA) and 1% penicillin-streptomycin solution (ScienCell). An incubator containing 5% CO_2_ at 37 °C provides a suitable environment for the cells.

### Human tissue samples

Shengjing Hospital of China Medical University provided us with human tissue samples. The samples comprised normal brain tissue (*n* = 9), low-grade glioma tissue (*n* = 9), and glioblastoma tissue (*n* = 9). All samples were collected after obtaining the signed consent of patients.

### Quantitative real-time PCR

Total RNA was extracted from cells or tissues using TRIzol reagent (Beyotime, Shanghai, China), and the concentration was measured using a Nanodrop Spectrophotometer (Thermo Fisher Scientific, USA). HiScript III RT SuperMix for qPCR and ChamQ Universal SYBR qPCR Master Mix (Vazyme, Nanjing, China) were used to detect RNA levels(using GAPDH as the internal reference). The primer sequences were listed in Supplementary Table 1.

### Western blot analysis

Total protein was harvested by cracking cells or tissues using RIPA and PMSF (Beyotime, Shanghai, China). The protein concentration was measured and balanced among different samples. Protein samples were subjected to SDS-PSGE (Beyotime, Shanghai, China) for electrophoretic separation and transferred onto PVDF membranes. Next, antibodies were added. A chemiluminescence imaging analysis system was used to measure spot intensity(Bio-Rad Laboratories, USA). Details of antibodies were listed in Supplementary Table 2.

### Cell transfection

When the growth density of the cells inoculated into the 24-well plates reached approximately 70%, we added the plasmid and Lipofectamine 3000 reagent. After incubation for 24 h, cells containing plasmid vectors were screened by adding the corresponding antibiotics according to the plasmid characteristics. The transfection efficiency was determined by quantitative real-time PCR (qPCR) and western blot analysis (WB). Full-length HECW1, ZNF350, JUND, and NCOA4 plasmids were constructed using the pcDNA 3.1. Small hairpin(sh)-HECW1, sh-ZNF350, sh-NCOA4 silencing plasmids were constructed using the pGPU6. The plasmids were purchased from Shanghai GenePharma. The plasmid sequences were listed in Supplementary Table 3.

### Chemicals

Ferrostain-1 (fer-1,), liproxstatin-1 (Lip-1,), erastin, RSL3, Z-VAD-FMK (Z-VAD), necrosulfonamide (NSA), necrostatin-1 (Nec-1), and MG-132 were purchased from GlpBio (Montclair, CA, USA). Cycloheximide (CHX) was purchased from Sigma-Aldrich (St. Louis, MO, USA). To induce ferroptosis, the concentration of erastin was diluted to 5 μmol/L and RSL3 to 2 μmol/L. The incubation time was 72 hours [40]. To inhibit apoptosis, ferroptosis, pyroptosis, or necroptosis, We treated the cells using Z-VAD (10 μmol/L), Fer-1 (1 μmol/L), Lip-1 (0.2 μmol/L), NSA (0.5 μmol/L), or Nec-1 (2 μmol/L) for 72 h [10, 41–43].

### Cell migration and invasion assay

In all, 200 μL serum-free medium containing 4 × 10^4^ cells was inoculated into the upper chamber of a polycarbonate membrane(Corning, New York, USA) with pore size of 8 μm, and 600 μl medium containing 10% fetal bovine serum(Gibco, California, USA) was added to the lower chamber. The cells were incubated for 48 h. Finally, after methanol fixation and 20% Jimsa staining, 5 randomly selected regions were counted and photographed under the microscope.

### Cell proliferation

Cell proliferation was measured using cell proliferation kit (BeyoClick™ EdU Cell Proliferation Kit; Beyotime, Shanghai, China). Cells were incubated at 10 µm EdU for 2 h and fixed with 4% paraformaldehyde. After washing off the fixative, PBS containing 0.3% Triton X-100 was added and incubated at room temperature for 10–15 min. CLICK reaction solution was added and incubated for 30 min in an environment protected from light. Hoechst 33342 was used for nuclear staining. Finally, fluorescence images of the EdU-incorporated samples were obtained using a fluorescence microscope.

### Cell viability assay

The CCK-8 kit was purchased from Beyotime (Shanghai, China). The glioma cells were seeded in 96-well plates at a density of 10,000 cells per well, 10 μL of CCK-8 solution was added to each well, and the plates were incubated for 1 h. Absorbance was then measured at 450 nm.

### lactate dehydrogenase measurements

Cell death was quantified by lactate dehydrogenase (LDH) release with an LDH assay kit (Abcam, Cambridge, UK). After centrifugation, the supernatants of cells were collected from the different treatment groups. Absorbance was measured at 450 nm.

### Lipid peroxidation measurements

Cellular LPO was quantified by measuring malondialdehyde (MDA) and hydrogen peroxide (H_2_O_2_) levels [10, 44]. The MDA assay kit and H_2_O_2_ assay kit were purchased from Beyotime (Shanghai, China). After the cells in each treatment group were completely lysed, the supernatant was collected(centrifugation: 12,000 × *g* /10 min). An appropriate amount of standard product was diluted with distilled water to 1, 2, 5, 10, 20, and 50 μM for the subsequent production of standard curves. The supernatant was mixed with the MDA working solution and heated at 100 °C for 15 min. After complete cooling, The mixture was centrifuged at 1000 ×*g* for 10 min. Finally, the absorbance was measured at 532 nm. We also measured the H_2_O_2_ levels, and the cells in each treatment group were harvested by centrifugation. The sample to be measured was obtained by adding the cracking solution and performing centrifugation again. After calibrating the reference standard of H_2_O_2_ and setting the standard curve, the absorbance at 560 nm was measured.

### Iron assay

Intracellular ferrous iron levels were quantified by colorimetric iron assay kit (Abcam). The supernatant was extracted by centrifugation after the cells from each treatment group were lysed using iron assay buffer. The iron probe was then added and incubated for 1 h. The absorbance was measured at 593 nm and the iron concentration was calculated.

### Protein degradation assay

CHX at a concentration of 10μmol/L was used to inhibit protein synthesis [45]. Cells were harvested after 0, 2, 4, and 6 h to extract proteins for WB analysis.

### Co-immunoprecipitation

The supernatant was extracted from the cells treated with NP-40 cell lysis buffer (Beyotime, Shanghai, China) after incubation, ultrasonic crushing, and centrifugation. The protein concentration of each group was measured and recorded. Cell lysates after antibody incubation were then incubated with A/G agarose beads(Santa Cruz, CA, USA). The immunoprecipitate was centrifuged to remove the supernatant and retain the beads. After adding 2xSDS-PAGE sample loading buffer (Beyotime, Shanghai, China) to the beads and boiling for 10 min, the prepared immune complexes were analyzed by WB.

### Ubiquitination assay

Cells were treated with 10 µM MG132 (Sigma-Aldrich, St. Louis, MO, USA) for 4–6 h. Immunoprecipitation was performed using whole-cell lysates. Lysis buffer containing phosphatase inhibitors (Beyotime, Shanghai, China), protease, and 10 mM *N*-ethylmaleimide (Sigma-Aldrich, St. Louis, MO, USA). Finally, the level of ZNF350 ubiquitination was detected by WB using an anti-Ub antibody.

### Chromatin immunoprecipitation

We purchased the EZ-ChIP™ kit for ChIP assay(Millipore, MA, USA). Proteins and DNA were crosslinked in 1% formaldehyde at room temperature for 10 min. The crosslinked chromatin was decomposed by ultrasound into DNA fragments of approximately 600 bp before adding normal rabbit IgG antibody or anti-ZNF350 antibody and incubating at 4 °C. After purification, the DNA fragments were amplified using 2X Tap Master Mix for PAGE (Vazyme, Nanjing, China), followed by agarose gel electrophoresis to verify the binding sites. The PCR primers are listed in Supplementary Table 4.

### Dual-luciferase reporter assays

The promoter region of NCOA4 was amplified from human genomic DNA by PCR, and the PCR products were subcloned into the pGL3 vector (Promega, Madison, WI, USA). Full-length human ZNF350 was constructed using the pEX3 (pGCMV/MCS/Neo) plasmid (GenePharma, Shanghai, China). The constructed vectors were transfected into HEK-293 T cells, and the relative luciferase activity was quantified as the ratio of firefly luciferase activity to Renilla luciferase activity.

### Tumor xenografts in nude mice

We purchased 4-week-old BALB/C athymic nude mice as test subjects. To exclude the influence of individual differences, U87 cells stably transfected with oeHECW1 were injected into the left underarm of nude mice, and U87 cells stably transfected with oeNC were injected into the right underarm of the same nude mice. After the tumor was clearly identified, the tumor volume (volume (mm^3^) = length × width ^2^/2) was regularly recorded on a 3-day cycle. After 42 days, all nude mice were sacrificed. The tumors were separated and weighed. In addition, tumor cells were implanted into the right striatum of mice through stereotaxis, and the survival number of nude mice was recorded. Finally, survival analysis was determined using Kaplan–Meier survival curve.

### Statistical analysis

GraphPad Prism8 and R Studio software were used for data analysis. Analysis of variance and Student’s t-tests were used to detect the differences in the results between groups. All values were presented as the mean ± standard deviation (S.D.) from at least three independent experiments (^NS^*P* > 0.05, *P < 0.05, **P < 0.01, ***P < 0.001 (In the study, “n” was used to represent the sample size or the number of experiments repeated)).

## Results

### HECW1, a potential prognostic biomarker, inhibited glioma cells progression

**Fig. 1:**
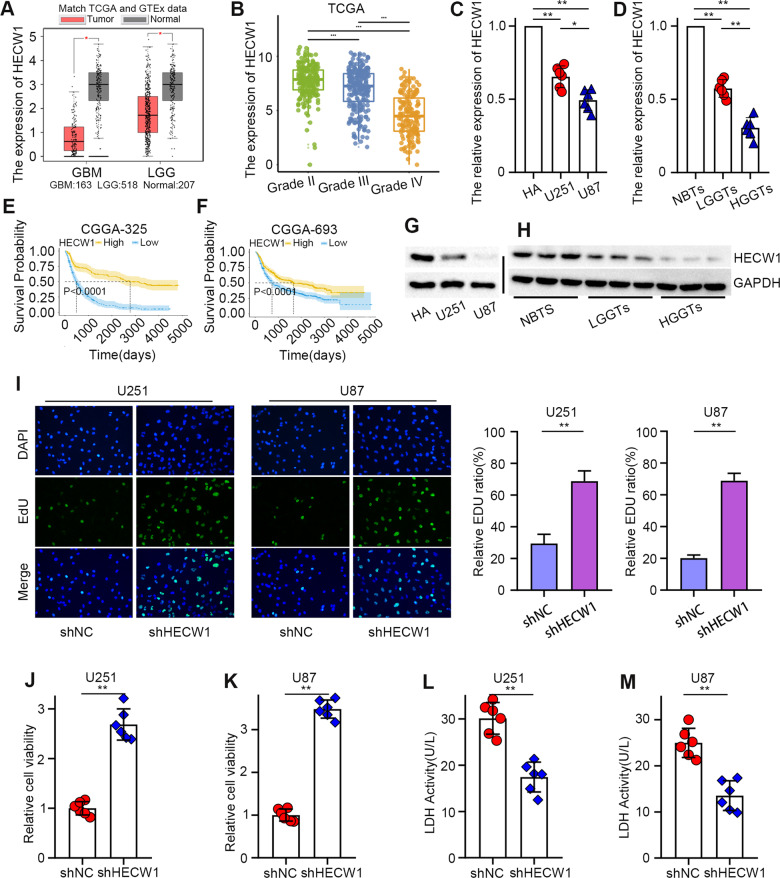
Expression levels of HECW1 and its effect on the growth of glioma cells. **A** Expression levels of HECW1 in bulk RNA sequencing data containing 163 GBM (TCGA), 518 LGG (TCGA), and 207 (GTEx) normal samples. **B** HECW1 expression is associated with glioma tumor grade. Data downloaded from the TCGA database. **C**, **G** HECW1 expression in HA, U251, and U87 cells detected by qPCR (*n* = 6) and WB analysis (*n* = 3). **E**, **F** Kaplan–Meier survival analysis. Data were downloaded from the CGGA database. CGGA325 and CGGA693 are two separate data sets. **D**, **H** Normal brain tissues (NBTs), low-grade glioma tissues (LGGTs), and high-grade glioma tissues (HGGTs) subjected to qPCR (*n* = 6) and WB analysis (*n* = 3) to detect HECW1 expression. **I** The proliferation of cells was detected using EDU kit (*n* = 3). **J**, **K** CCK8 assay used to detect the change of glioma cell viability after HECW1 knockdown (*n* = 6). **L**, **M** Cell death analyzed by measuring LDH release (*n* = 6). All values are presented as the mean ± S.D., *P < 0.05, **P < 0.01, ***P < 0.001.

Bioinformatics analysis indicated that HECW1 was mainly enriched in normal tissues (Fig. 1A) and decreased with the increase of glioma grade (Fig. 1B). The qPCR results revealed that HECW1 mRNA levels in human astrocytes (HA) were distinctly higher than those in U251 and U87 cells (Fig. 1C). Moreover, HECW1 expression was highest in peritumoral normal brain tissues and was significantly negatively correlated with the degree of tumor malignancy (Fig. 1D). Survival analysis (*n* = 325, data sources: CGGA) showed that high HECW1 expression predicted a more favorable prognosis for patients with glioma (Fig. 1E). Another survival analysis (*n* = 693; data source: CGGA) confirmed the predictive effect of high HECW1 expression on longer survival times in patients (Fig. 1F). WB analysis confirmed that HECW1 expression trend in the same cells and tissues was approximately consistent with the qPCR results (Fig. 1G, H). We constructed stable transfected cells and measured HECW1 protein levels by WB analysis (Supplementary Fig. 1A). Figure 1I confirmed that the proliferation capacity of HECW1-deficient cells was greatly enhanced. Furthermore, HECW1 knockdown significantly enhanced glioma cell activity (Fig. 1J, K). This decrease in LDH release confirmed that HECW1 knockdown reduced glioma cell death (Fig. 1L, M). Transwell assays demonstrated that HECW1-deficient cells acquired stronger migration and invasion ability (Supplementary Fig. 1B). According to these results, we conclude that high HECW1 expression predicts better prognosis and more favorable tumor grading in patients with glioma. The deficiency of HECW1 accelerates the malignant progression of glioma cells.

### HECW1 exerts tumor-suppressive function by inducing ferroptosis in glioma cells

**Fig. 2:**
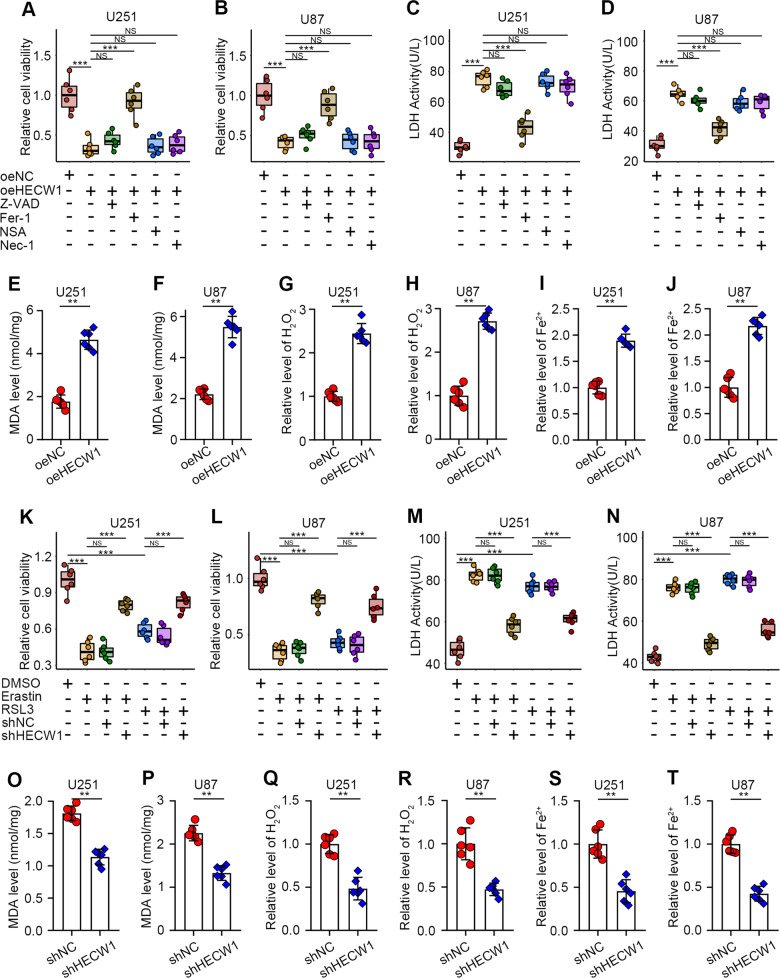
HECW1 induced ferroptosis in glioma cells. **A**, **B** After 72 hours of drug treatment, cell viability was determined by CCK8 assay (*n* = 6). **C**, **D** LDH release levels after 72 h of treatment with various inhibitors (*n* = 6). **E**, **F** Quantification of MDA levels (*n* = 6). **G**, **H** Intracellular H2O2 levels (*n* = 6). **I**, **J** Determination of ferrous iron levels in glioma cells (*n* = 6). **K**–**N** Tolerance of HECW1-deficient cells to ferroptosis inducers was determined by CCK8 and LDH release levels (*n* = 6). **O**–**T** Intracellular MDA, H2O2, and ferrous iron levels(*n* = 6). All values are presented as the mean ± S.D., ^NS^*P* > 0.05, *P < 0.05, **P < 0.01, ***P < 0.001.

To uncover the mechanism by which HECW1 plays a role in cancer inhibition, we treated glioma cells with different cell death inhibitors. Firstly, we determined the protein level of HECW1 in cells transfected with oeHECW1 by WB analysis (Supplementary Fig. 2A). HECW1 overexpression markedly reduced glioma cell growth (Fig. 2A, B). Interestingly, this effect was reversed by ferroptosis inhibitors (Fer-1; Fig. 2A, B). Moreover, overexpression of HECW1 significantly increased glioma cell death, which was largely reversed by Fer-1 treatment (Fig. 2C, D). In further experiments, we verified that the overexpression of HECW1 markedly improved MDA, intracellular ROS (H_2_O_2_), and ferrous iron levels (Fig. 2E–J). These three are the major factors that lead to LPO and ferroptosis in cells. Next, we revealed the effect of HECW1 knockdown on ferroptosis. As expected, ferroptosis inducers (erastin, RSL3) significantly inhibited glioma cell growth and accelerated cell death; however, this effect was largely offset by HECW1 silencing (Fig. 2K–N). The three key inducers of ferroptosis also decreased significantly after HECW1 silencing (Fig. 2O–T). Our results indicated that HECW1 causes iron accumulation, LPO, and ferroptosis in glioma cells.

### HECW1 activates ferroptosis by positively regulating NCOA4

**Fig. 3:**
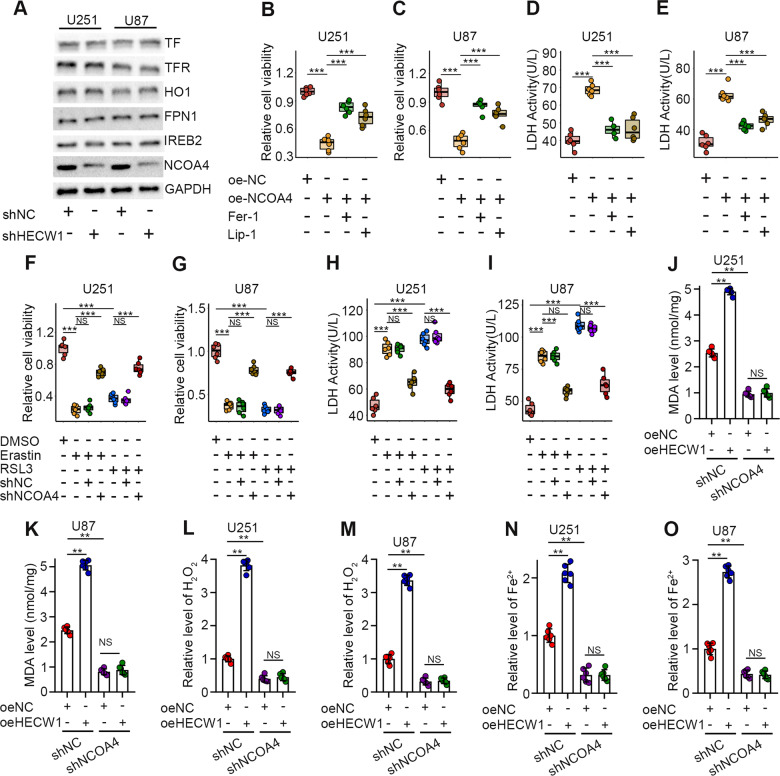
HECW1 overexpression promoted NCOA4-mediated ferroptosis. **A** Change of candidate protein expression after HECW1 knockdown determined by WB (*n* = 3). **B**–**E** Cell activity and LDH release levels measured 72 h after the addition of ferroptosis inhibitors (Fer-1,Lip-1; *n* = 6). **F**–**I** Tolerance of NCOA4-deficient cells to ferroptosis inducers was determined by CCK8 and LDH release levels(*n* = 6). **J**, **K** Quantification of MDA levels(*n* = 6). **L**, **M** Intracellular H2O2 levels(*n* = 6). **N**, **O** Determination of ferrous iron levels in glioma cells. All values are presented as the mean ± S.D., ^NS^*P* > 0.05, *P < 0.05, **P < 0.01, ***P < 0.001.

We explored the mechanism by which HECW1 regulates ferroptosis. HECW1, an E3 ubiquitin ligase, likely plays a role in regulating the ubiquitination and degradation of target proteins. Therefore, we screened for ferroptosis-related proteins and observed changes in their expression in HECW1-silenced cells. WB analysis revealed that only NCOA4 protein expression was significantly altered in HECW1-silenced glioma cells (Fig. 3A). Next, we examined whether NCOA4 acts as a tumor suppressor in gliomas by eliciting ferroptosis. Similarly, we measured protein levels of NCOA4 in stable transfected cells (Supplementary Fig. 3A, B). Compared with that in the negative control group, the growth of glioma cells in the NCOA4 overexpression group was greatly inhibited, whereas cell death was notably increased (Fig. 3B–E). However, the cancer-inhibiting effect of NCOA4 overexpression was severely disrupted by ferroptosis inhibitors (Fig. 3B–E). As shown in Fig. 3F–I, NCOA4 silencing largely counteracts the toxicity of ferroptosis inducers to glioma cells. Therefore, we conclude that NCOA4 acts as a driver of ferroptosis in glioma cells. Supplementary Fig. 3C, D demonstrated that overexpression of HECW1 could not exert tumor inhibitory effect in NCOA4-deficient glioma cells. As shown in Fig. 3J–O, under the premise of not interfering with the expression of NCOA4, the three indexes of ferroptosis in HECW1-overexpressed cells were distinctly boosted, which was consistent with our previous experimental results. Encouragingly, after NCOA4 silencing, we observed no significant difference in the three ferroptosis indexes between the HECW1 overexpression group and negative control groups (Fig. 3J–O). These findings suggest that HECW1 induces ferroptosis through the positive regulation of NCOA4.

### ZNF350, a potential predictor of glioma prognosis, mediates the positive regulation of NCOA4 by HECW1

**Fig. 4:**
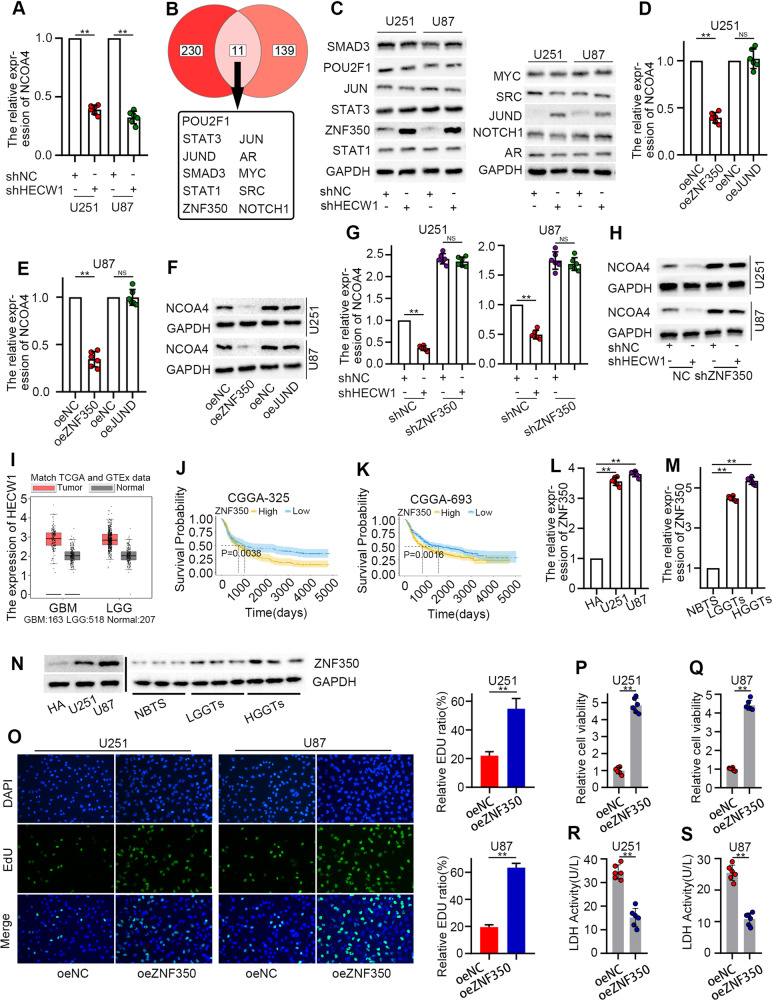
ZNF350 accelerated glioma cells growth and mediated the positive regulation of NCOA4 by HECW1. **A** NCOA4 expression in HECW1-silenced cells and control cells determined by qPCR (*n* = 6). **B** Intersection of HECW1 ubiquitination substrates predicted by the UbiBrowser database (top 150 genes in descending order of confidence score) and transcription factors regulating NCOA4 predicted by the HumanTFDB database (*P* < 0.05). **C** Expression of candidate proteins detected by WB (*n* = 3). **D**, **E** Effect of ZNF350 and JUND overexpression on the transcription level of NCOA4 detected by qPCR (*n* = 6). **F** Effect of ZNF350 and JUND overexpression on the translation level of NCOA4 detected by WB (*n* = 3). **G** NCOA4 expression detected by qPCR (*n* = 6). **H** Protein levels of NCOA4 detected by WB analysis (*n* = 3). **I** ZNF350 expression in bulk RNA sequencing data, which contained 163 GBM (TCGA), 518 LGG (TCGA), and 207 normal (GTEx) samples. **J**, **K** Kaplan–Meier survival analysis. Data were downloaded from the CGGA database. CGGA325 and CGGA693 are two separate data sets. **L**, **N** ZNF350 expression in HA, U251, and U87 cells detected by qPCR (*n* = 6) and WB (*n* = 4). **M**, **N** ZNF350 expression in NBTs, LGGTs, and HGGTs detected by qPCR (*n* = 6) and WB (*n* = 4). **O** EDU kit was used to detect cell proliferation (*n* = 3). **P**–**S** Cell viability and LDH levels of ZNF350 overexpression cells and control cells (*n* = 6). All values are presented as the mean ± S.D., ^NS^*P* > 0.05, *P < 0.05, **P < 0.01, ***P < 0.001.

An interesting phenomenon is observed whereby the protein expression of NCOA4 decreased after HECW1 silencing (Fig. 3A). Similarly, HECW1 knockdown significantly downregulated the mRNA level of NCOA4 (Fig. 4A). Therefore, we speculate that HECW1 regulates NCOA4 not by directly mediating NCOA4 ubiquitination, but by influencing the transcription level of NCOA4. Figure 4B shows the intersection of the potential ubiquitination substrate protein of HECW1 with the transcription factors that regulate NCOA4 expression. Protein levels of both ZNF350 and JUND were upregulated in HECW1-deficient glioma cells (Fig. 4C). However, the overexpression of ZNF350 significantly downregulated the mRNA and protein levels of NCOA4, unlike JUND treatment (Fig. 4D–F). Our experiments demonstrated that HECW1 knockdown notably inhibited the transcription and translation of NCOA4. However, this effect disappeared after ZNF350 silencing (Fig. 4G, H). The supplementary Fig. 4B suggested that ZNF350 overexpression completely blocked the positive regulation of NCOA4 by HECW1 overexpression. We found that ZNF350 was enriched in GBM and LGG tissues (Fig. 4I). Survival analysis involving a large number of glioma samples revealed that ZNF350 low expression group had higher survival rate (Fig. 4J, K). Subsequent experiments demonstrated that ZNF350 expression was highest in U87 cells and lowest in HA cells (Fig. 4L, N). Similarly, ZNF350 expression was significantly higher in glioma tissues than in normal brain tissues and higher in HGGTs than in LGGTs (Fig. 4M, N). Figure 4O demonstrated that overexpression of ZNF350 significantly promoted the proliferation of glioma cells. Transwell assay indicated that ZNF350 overexpressed cells had stronger migration and invasion ability(Supplementary Fig. 4A). Our experiments also showed that ZNF350 overexpression promoted glioma cell viability and inhibited cell death (Fig. 4P–S). These results suggested that ZNF350 is a potential predictor of poor prognosis in patients with glioma, greatly promotes the growth of glioma cells in vitro, and mediates the positive regulation of HECW1 on NCOA4.

### HECW1 acts as a ferroptosis inducer by controlling ubiquitination and degradation of ZNF350

**Fig. 5:**
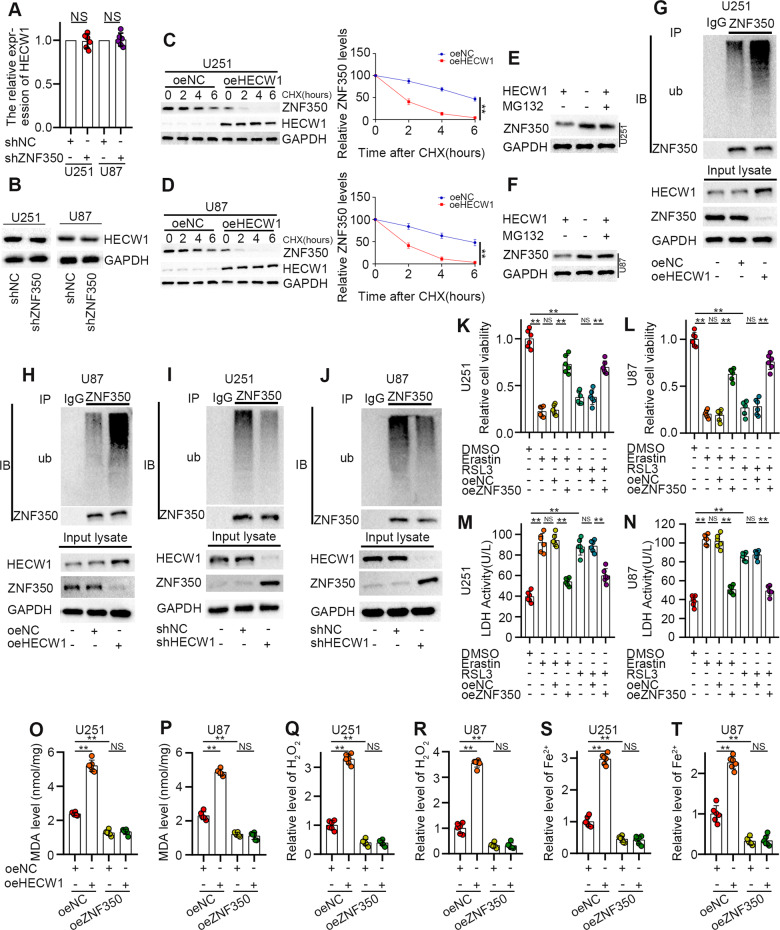
HECW1 mediated ubiquitination and degradation of ZNF350 to induce ferroptosis. **A**, **B** HECW1 expression detected by qPCR (*n* = 6) and WB (*n* = 6). **C**, **D** The protein levels of ZNF350 at different time periods were determined by WB analysis (*n* = 3). **E**, **F** ZNF350 protein levels quantified by WB (MG132 is a proteasome inhibitor *n* = 3). **G**–**J** Ubiquitination level of ZNF350 measured after HECW1 knockdown and overexpression (*n* = 3). **K**–**N** Tolerance of ZNF350 overexpression cells to ferroptosis inducers was determined by CCK8 and LDH release levels(*n* = 6). **O**, **P** Quantification of MDA levels (*n* = 6). **Q**, **R** Intracellular H2O2 levels (*n* = 6). **S**, **T** Determination of ferrous iron levels in glioma cells (*n* = 6). All values are presented as the mean ± S.D., ^NS^*P* > 0.05, *P < 0.05, **P < 0.01, ***P < 0.001.

Next, we investigated the specific mechanism by which HECW1 negatively regulates ZNF350 expression. As a transcription factor, ZNF350 did not affect HECW1 expression in glioma cells (Fig. 5A, B). When protein synthesis in the cells was inhibited by CHX, we detected changes in ZNF350 protein levels at four different time points. Specifically, the degradation rate of ZNF350 increased noticeably after HECW1 expression was enhanced (Fig. 5C, D). However, the proteasome inhibitor MG132 reversed this decline and enhanced the stability of ZNF350 (Fig. 5E, F), suggesting that ZNF350 was degraded by the ubiquitin-proteasome pathway. Quantification of ubiquitin levels confirmed this finding. After the immunoprecipitation of ZNF350 from MG132-treated cells, ZNF350 was heavily ubiquitinated and degraded in the HECW1-overexpression group, whereas ZNF350 ubiquitination was severely inhibited and ZNF350 stability was greatly enhanced in the HECW1-knockdown group (Fig. 5G–J). To determine the association between ZNF350 and ferroptosis, glioma cells that were stably transfected with oeZNF350 and oeNC were treated with Erastin or RSL3. As expected, overexpression of ZNF350 not only substantially downregulated cell loss caused by ferroptosis inducers, but also counteracted the inhibitory effect of ferroptosis inducers on cell viability (Fig. 5K–N). In addition, we demonstrated that ZNF350 overexpression reversed the HECW1-induced accumulation of MDA, ROS, and Fe^2+^ in glioma cells (Fig. 5O–T). These data imply that high ZNF350 expression can help glioma cells escape ferroptosis and that HECW1-mediated ubiquitination and degradation of ZNF350 is a crucial mechanism for HECW1-induced ferroptosis.

### ZNF350 directly inhibits NCOA4 transcription to resist ferroptosis

**Fig. 6:**
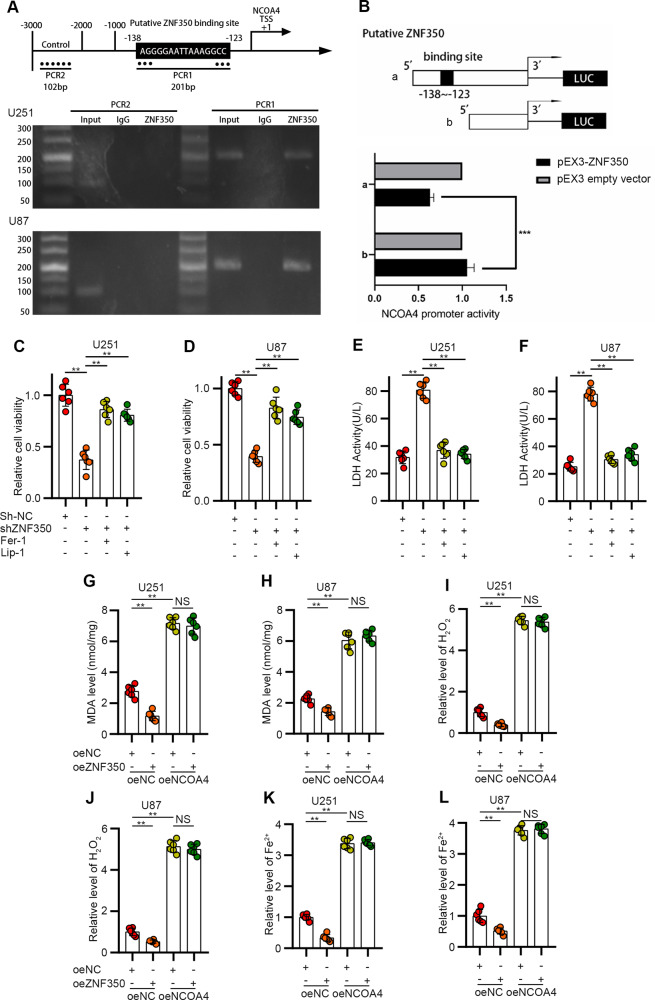
ZNF350 inhibited NCOA4-regulated ferroptosis by directly downregulating NCOA4 transcription. **A** Binding sites of ZNF350 on the promoter region of NCOA4 determined by ChIP assays. Immunoprecipitated DNA was amplified by primers represented by dashed lines. Transcription start site (TSS) was designated as +1. Normal IgG was the negative control and −2000 to −3000 was the blank control. **B** Schematic depiction of different reporter vectors and relative luciferase activity of NCOA4 (*n* = 3). **C**–**F** Cell activity and LDH release levels measured 72 h after the addition of ferroptosis inhibitors (Fer-1, Lip-1) (*n* = 6). **G**, **H** Quantification of MDA levels (*n* = 6). **I**, **J** Intracellular H2O2 levels (*n* = 6). **K**, **L** Determination of ferrous iron levels in glioma cells (*n* = 6). All values are presented as the mean ± S.D., ^NS^*P* > 0.05, *P < 0.05, **P < 0.01, ***P < 0.001.

The chromatin immunoprecipitation (ChIP) assay demonstrated that ZNF350 bound to the NCOA4 promoter region at sites 138 and 123 (Fig. 6A). The fluorescence activity of pEX3-ZNF350 was markedly decreased in luciferase reporter assays (Fig. 6B). We further verified the inhibitory effects of ZNF350 on ferroptosis. We observed that the two independent ferroptosis inhibitors blocked cell death and decreased cell viability induced by ZNF350 knockdown (Fig. 6C–F). According to these results and those in Fig. 5K–N, we concluded that ZNF350 acts as a ferroptosis suppressor in glioma cells. To confirm that ZNF350 resistance to ferroptosis depends on the transcriptional inhibition of NCOA4, we constructed ZNF350 and NCOA4 double-transfected cells. As shown in Fig. 6G–L, the overexpression of NCOA4 completely reversed the downregulation of MDA, ROS, and Fe^2+^ levels caused by the overexpression of ZNF350. Similarly, knocking down NCOA4 eliminated the upregulatory effect of ZNF350 silencing on MDA, ROS, and Fe^2+^, and even maintained the levels of these three indexes at low levels (Supplementary Fig. 6A–F). These findings demonstrate that ZNF350 can inhibit the transcription of NCOA4 by directly binding to the promoter region of NCOA4, acting as a ferroptosis suppressor in glioma cells. Taken together, we confirmed that HECW1, ZNF350, and NCOA4 form an integral pathway involved in the regulation of ferroptosis in gliomas.

### HECW1 functions as a novel tumor suppressor in vivo

**Fig. 7:**
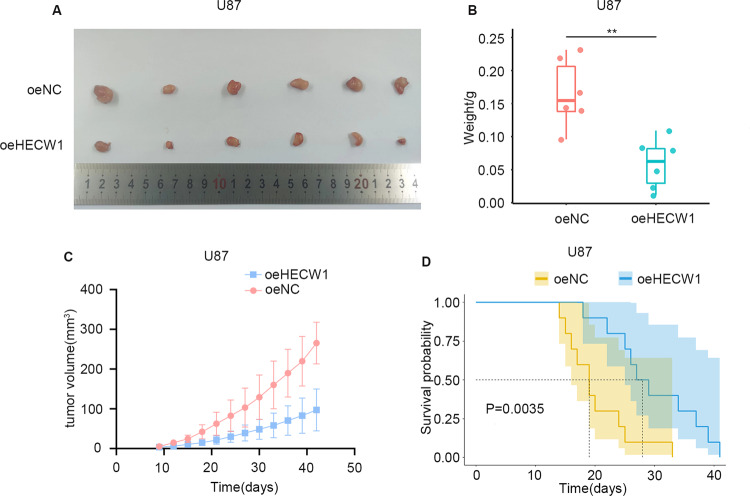
HECW1 overexpression inhibited tumor growth and improved survival rate in nude mice. **A** Final tumor volumes in the oeNC and oeHECW1 groups (*n* = 6). **B** Boxplot showed the final tumor weights of the oeNC and oeHECW1 groups**. C** The growth curve recorded the volume change of tumors**. D** Kaplan–Meier method was used for survival analysisn(*n* = 10). All values are presented as the mean ± S.D., ^NS^*P* > 0.05, *P < 0.05, **P < 0.01, ***P < 0.001.

To investigate the biological effects of HECW1 in vivo, We constructed xenograft tumor model in nude mice. The result indicated that the tumor volume in oeHECW1 group was significantly smaller than that in oeNC group (Fig. 7A). Figure 7B, C suggested that the tumor weight and growth rate of oeHCEW1 group were effectively inhibited. Survival analysis confirmed that nude mice in the oeHECW1 group had a longer survival time (Fig. 7D). These findings confirmed that HECW1 exerted a powerful tumor suppressant effect in vivo.

## Discussion

Glioma is an aggressive brain tumor that accounts for 80% of central nervous system malignancies [2, 46]. Despite tremendous efforts, the overall survival time of patients with glioblastoma is no longer than two years [47].

Ubiquitination is a complex mechanism of post-translational protein modification involving a large number of biological processes, including proteasome degradation, protein–protein interactions, DNA repair, and gene transcription [48, 49]. The binding of ubiquitin to its substrate, mediated by E3 ubiquitin ligase, is the most critical process in ubiquitination [50]. Various E3 ubiquitin ligases play important roles in the development and progression of tumors by regulating the degradation of oncogenes or their interactions with tumor suppressors [51]. Therefore, ubiquitination signal targeting based on E3 ubiquitin ligase is a promising strategy for tumor treatment. HECW1, an E3 ubiquitin ligase, is involved in the regulation of non-small cell lung cancer, breast cancer, and thyroid cancer by mediating the ubiquitination and degradation of target proteins [23, 26, 52]. In this study, HECW1 expression was severely downregulated in glioma cells and tissues. Higher HECW1 expression was associated with longer overall survival in patients with glioma. Cells with HECW1 deficiency showed significantly increased proliferation, migration, invasion, and activity and markedly decreased mortality, which is consistent with the characteristics of the tumor suppressor gene. Through the use of cell death inhibitors and ferroptosis inducers, as well as the determination of intracellular ferrous and LPO indices, we demonstrated that HECW1 is involved in intracellular iron metabolism and ferroptosis.

Drug resistance has always been a major obstacle to the success of tumor-targeted therapy [53]. However, resistant cells, especially those in the mesenchymal and dedifferentiated states of tumor cells, are susceptible to ferroptosis, which underscores the potential of ferroptosis in eradicating tumors [54, 55]. Intracellular iron mediates the generation of toxic lipid-based ROS through the Fenton reaction and induces ferroptotic cell death [56–58]. Therefore, dysregulation of iron homeostasis is a key metabolic marker of cellular ferroptosis. Transferrin (TF), an important iron transport protein, binds to the membrane protein transferrin receptor (TFR) to form a complex [59]. Iron absorption in most cells occurs via endocytosis, which is triggered by the TF/TFR complex [60]. Moreover, heme oxygenase-1 can change the distribution of iron in cells by catalyzing the degradation of heme to release free iron [61, 62]. The only known iron-exporting protein in mammals, ferroportin1, exports iron across the basolateral membrane [63, 64]. Therefore, the downregulation of ferroportin1 can induce ferroptosis by promoting iron accumulation [63]. Furthermore, IREB2 plays an important role in cellular iron homeostasis by regulating the stability of iron transporters via post-transcriptional mechanisms [65]. Lastly, NCOA4 acts as a cargo receptor to mediate the selective autophagic degradation of ferritin, known as ferritinophagy, which results in the leakage of Fe^2+^ into the cytoplasm, leading to ferroptosis [66, 67]. Here, we found that only NCOA4 protein expression was significantly downregulated after HECW1 silencing, whereas the expression of TF, TFR, heme oxygenase-1, ferroportin1, and IREB2 did not change. NCOA4 knockdown blocked ferroptosis induced by HECW1 overexpression. Interestingly, after HECW1 silencing, the transcription level of NCOA4 was also dramatically downregulated. Therefore, we hypothesized that HECW1 induces iron accumulation, LPO, and ferroptosis, not directly through the ubiquitination and degradation of NCOA4, but by affecting the transcription of NCOA4. Specifically, we demonstrated that ZNF350 mediates the positive regulation of NCOA4 by HECW1.

ZNF350, a transcriptional suppressor, binds to BRCA1 and CtIP to form a transcriptional suppressor complex that inhibits angiogenesis in breast cancer [33]. BRCA1 and ZBRK1 then form a complex that inhibits breast cancer cell proliferation [27]. Several studies have shown that ZNF350 can inhibit transcription as a monomer. For example, ZNF350 inhibits cervical cancer progression by directly binding to the MMP9 promoter region and inhibiting its transcription [30]. In this study, we found that ZNF350 was upregulated in gliomas and predicted a shorter survival time in patients. We also confirmed that ZNF350 not only promotes glioma cell proliferation, migration, invasion, and activity but also reduces cell death. The main reason for this phenomenon was ferroptosis inhibition by ZNF350 in glioma cells. As expected, protein degradation and ubiquitination assays revealed that HECW1, an E3 ubiquitin ligase, controlled the ubiquitination and degradation of ZNF350. ChIP and dual-luciferase gene reporter assays confirmed that ZNF350 inhibited NCOA4-mediated iron accumulation, LPO, and ferroptosis by downregulating NCOA4 transcription levels through direct binding to the promoter region of NCOA4 in the monomer form.

In summary, this study is the first to reveal the influence of HECW1 and ZNF350 on gliomas and their potential value in evaluating patient prognosis. More importantly, we explore and demonstrate the role and mechanism of the HECW1/ZNF350/NCOA4 pathway in regulating ferroptosis and identify new molecular targets for glioma therapy.

### Supplementary information

SUPPLEMENTARY MATERIALS

Original Data File

Reproducibility Checklist

## Data Availability

Data not included in the Supplementary Material can be obtained from the corresponding author.
