# Supplementary material for: HECW1 induces NCOA4-regulated ferroptosis in glioma through the ubiquitination and degradation of ZNF350
Source: Cell Death Dis. 2023 Dec 4;14(12):794. doi: 10.1038/s41419-023-06322-w (PMC10695927; doi:10.1038/s41419-023-06322-w)
Supplement: Supplementary file 1 — SUPPLEMENTARY MATERIALS [file 41419_2023_6322_MOESM1_ESM.docx]

**Supplementary Table 1:Primer sets used for RT-qPCR**

| **Gene ID** | **Forward** | **Reverse** |
| --- | --- | --- |
| HECW1 | GTTTTGTGTCCTTGCCCACT | GAATTGCAGCTGTCCACTCA |
| ZNF350 | TCTTGTGTATCTGGAGAAAATAGAGGT | AAGAAATGGTGAACCCCAAA |
| NCOA4 | CAGCAGCTCTACTCGTTATTGG | TCTCCAGGCACACAGAGACT |
| GAPDH | CAAGGCTGAGAACGGGAAG | TGAAGACGCCAGTGGACT |

**Supplementary Table 2:Details of antibodies**

| **Gene ID** | **CAT.NO** | **Manufacturer** |
| --- | --- | --- |
| HECW1 | #24695-1-AP | Protentec |
| ZNF350 | #ab77085 | Abcam |
| NCOA4 | #ab62495 | Abcam |
| TF | #17435-1-AP | Protentec |
| TFR | #10084-2-AP | Protentec |
| HO1 | #10701-1-AP | Protentec |
| FPN1 | #26601-1-AP | Protentec |
| IREB2 | #23829-1-AP | Protentec |
| SMAD3 | #66516-1-Ig | Protentec |
| POU2F1 | #10387-1-AP | Protentec |
| MYC | #67447-1-Ig | Protentec |
| STAT1 | #10144-2-AP | Protentec |
| JUN | #ab40766 | Abcam |
| SRC | #11097-1-AP | Protentec |
| JUND | #61404 | Protentec |
| NOTCH1 | #ab52627 | Abcam |
| AR | #22089-1-AP | Protentec |
| STAT3 | #10253-2-AP | Protentec |
| IgG | #30000-0-AP | Protentec |
| GAPDH | #60004-1-Ig | Protentec |

**Supplementary Table 3: Plasmid sequences**

| **Plasmid ID** | **Sequence** |
| --- | --- |
| sh-HECW1 | GACCTCACTTTCACTGTTAAT |
| sh-ZNF350 | GAAAUCAGGUCUCAUUAAA |
| sh-NCOA4 | CTCTTATTCCAGTCCTATAAT |
| sh-NC | TTCTCCGAACGTGTCACGT |

**Supplementary Table 4: Primer sets used for CHIP**

| **Plasmid ID** | **Sequence** |
| --- | --- |
| PCR 1 L | TCCTCTGTGTTTGCTTGAGGT |
| PCR 1 R | TGTAGCAAGAGGTAGCTGATGC |
| PCR 2 L | AGACAAAACTACATATGAATGGCCG |
| PCR 2 R | CGATCCCCTGACCTCAAATGA |

**Supplementary Figure1**

**
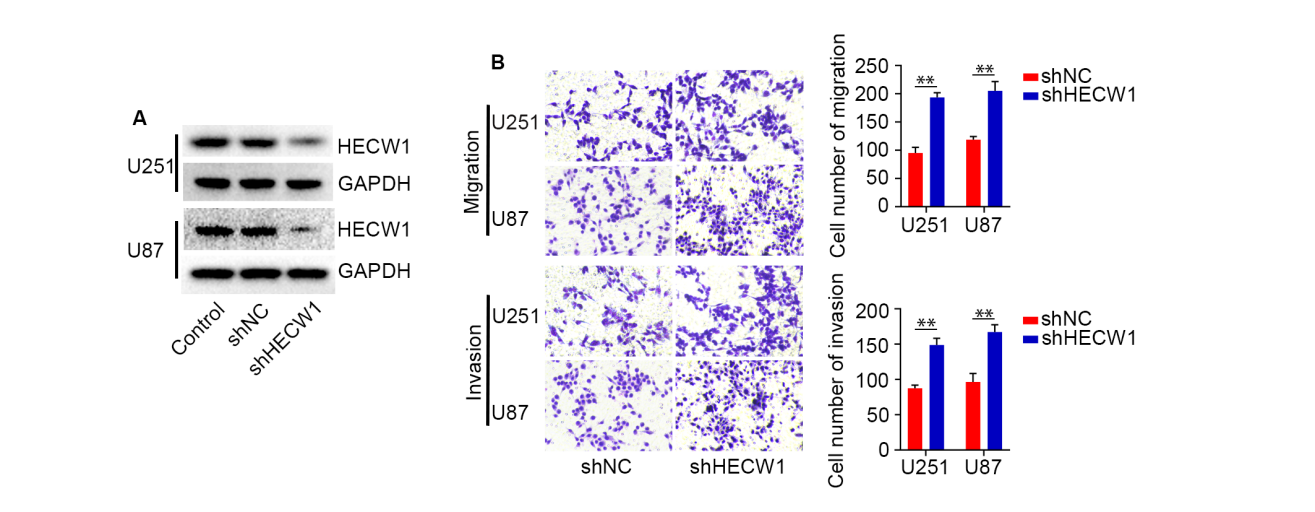
**

**Supplementary Figure1. Expression levels of HECW1 in glioma tissues and cell lines and their effect on glioma cells.** **(A)** Protein levels of HECW1 were determined by WB(n=3). **(B)** Transwell assay quantified the effect of HECW1 on migration and invasion ability of glioma cells(n=3) .All values are presented as the mean ± S.D., * P < 0.05, ** P < 0.01, *** P < 0.001.

**Supplementary Figure2**


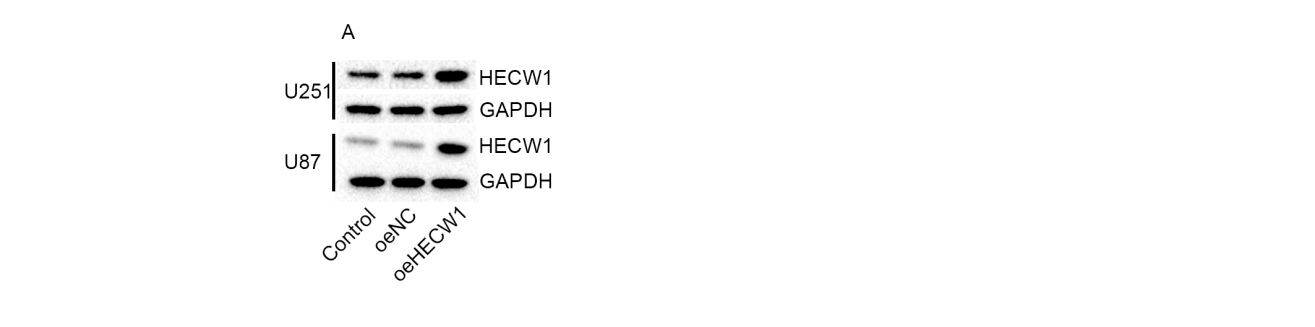


**Supplementary Figure2. HECW1 induced ferroptosis in glioma cells.** **(A)** Protein levels of HECW1 were determined by WB(n=3).

**Supplementary Figure3**

**
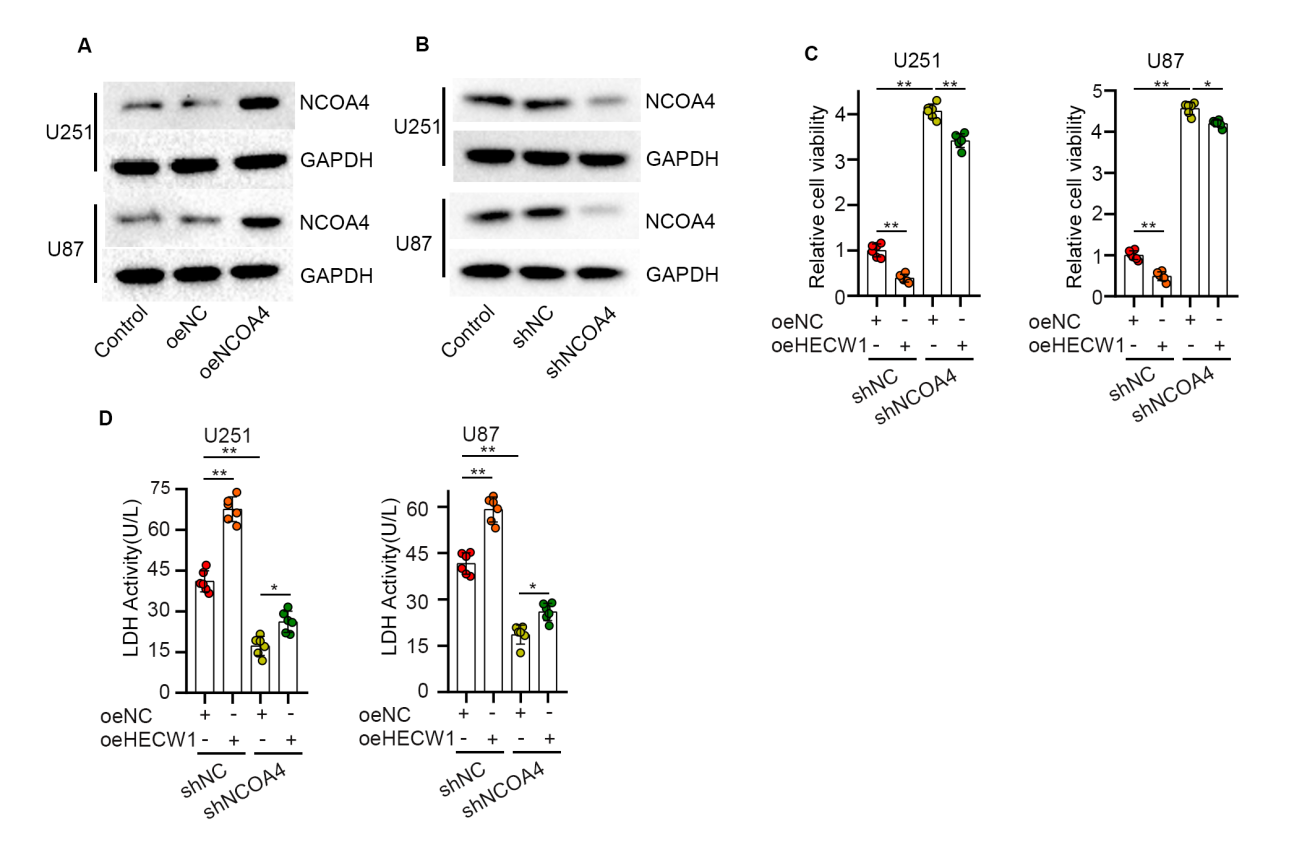
**

**Supplementary Figure3.HECW1 overexpression promoted NCOA4-mediated ferroptosis.(A,B)** Protein levels of NCOA4 in stably transfected cells was determined by WB (n=3). **(C,D)** Cell activity and LDH release levels were measured (n=6). All values are presented as the mean ± S.D., ^NS^ P > 0.05, * P < 0.05, ** P < 0.01, *** P < 0.001.

**Supplementary Figure4**

**
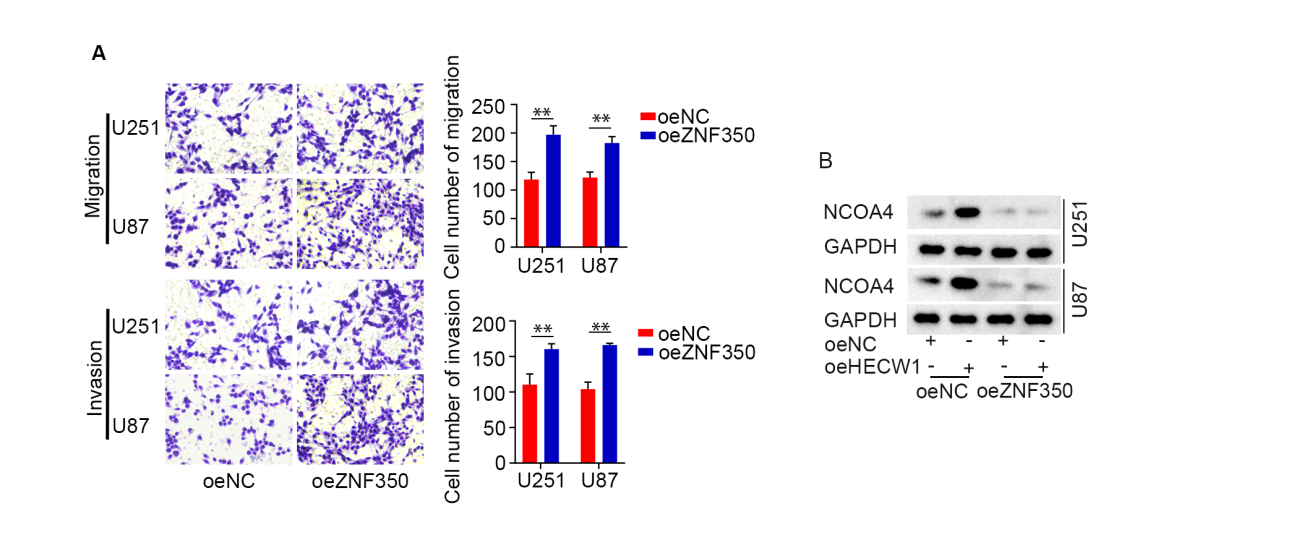
**

**Supplementary Figure4.ZNF350 accelerated glioma cells growth and mediated the positive regulation of NCOA4 by HECW1.(A)** Transwell assay quantified the effect of ZNF350 on migration and invasion ability of glioma cells(n=3). **(B)** The protein levels of NCOA4 were determined by WB analysis(n=3). All values are presented as the mean ± S.D., ^NS^ P > 0.05, * P < 0.05, ** P < 0.01, *** P < 0.001.

**Supplementary Figure6**

**
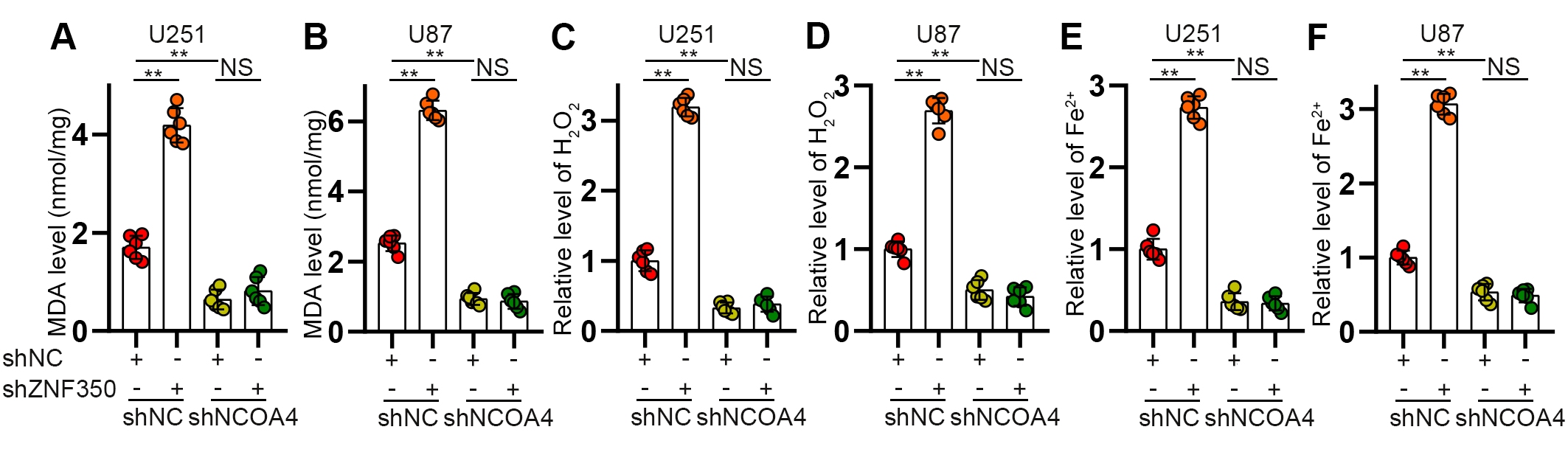
**

**Supplementary Figure 6. ZNF350 inhibits NCOA4-regulated ferroptosis by directly down-regulating NCOA4 transcription.(A,B)** Quantification of MDA levels(n=6). **(C,D)** Intracellular H2O2 levels(n=6). **(E,F)** Determination of ferrous iron levels in glioma cells(n=6).All values were presented as the mean ± S.D.,^NS^P>0.05 ,*P < 0.05,**P<0.01, ***P<0.001.

**Certificate_of_editing-SJING_1875_hykfbqkqxj**

**
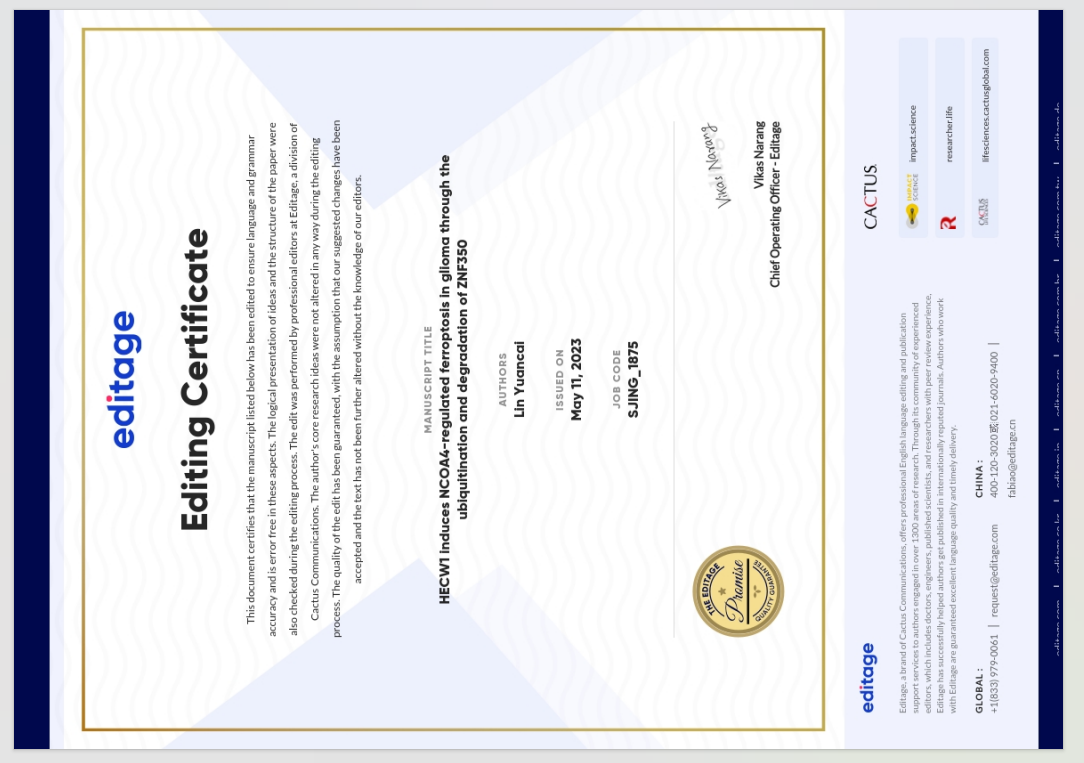
**
