# Supplementary material for: HECW1 induces NCOA4-regulated ferroptosis in glioma through the ubiquitination and degradation of ZNF350
Source: Cell Death Dis. 2023 Dec 4;14(12):794. doi: 10.1038/s41419-023-06322-w (PMC10695927; doi:10.1038/s41419-023-06322-w)

Figure1 G


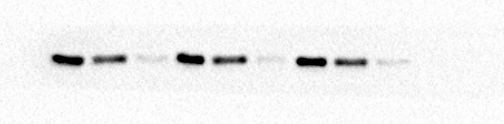


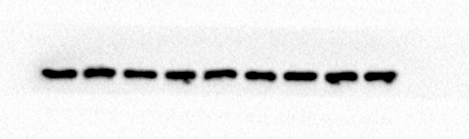


Figure1 H


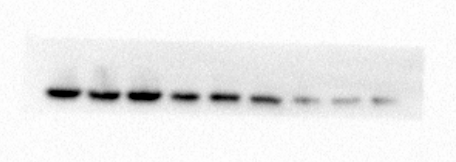


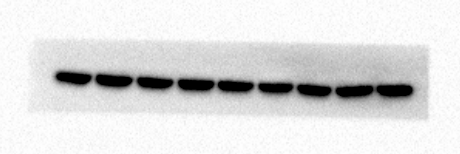


**Supplementary Figure1 A(U251)**


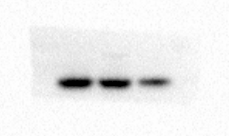


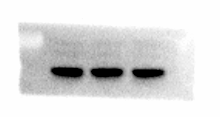


**Supplementary Figure1 A(U87)**

**
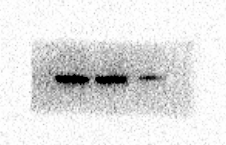
**


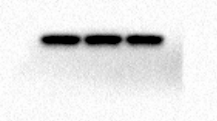


**Supplementary Figure2 A(U251)**


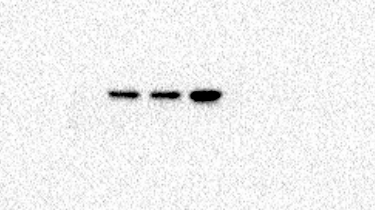


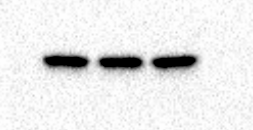


**Supplementary Figure2 A(U87)**


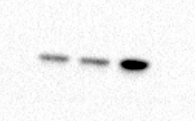


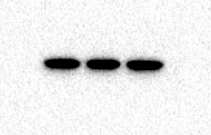


**Supplementary Figure3 A(U251)**


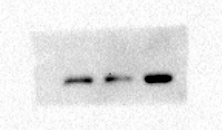


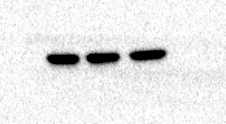


**Supplementary Figure3 A(U87)**


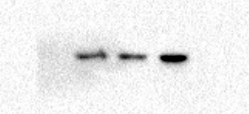


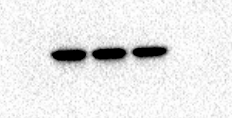


**Supplementary Figure3 B(U251)**


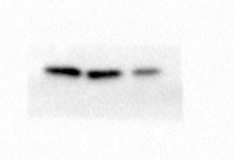


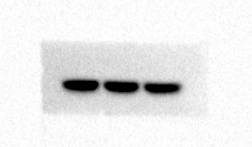


**Supplementary Figure3 B(U87)**


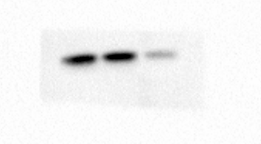


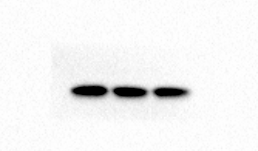


Figure3 A


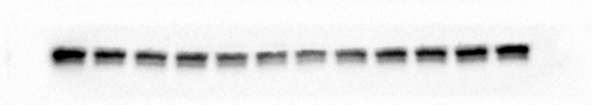


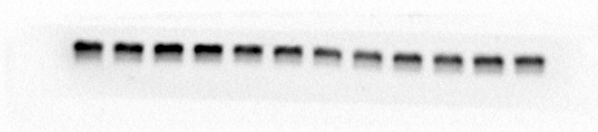


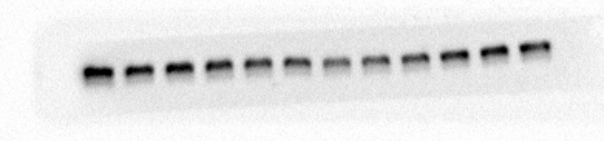


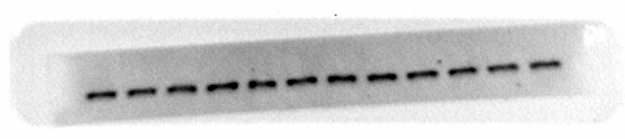


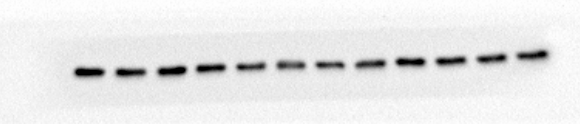


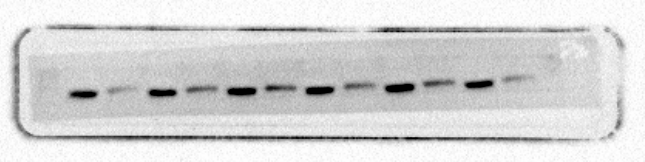


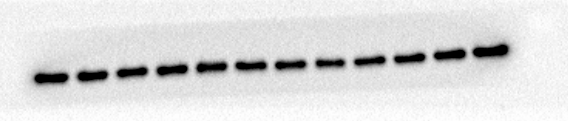


Figure4 C

left


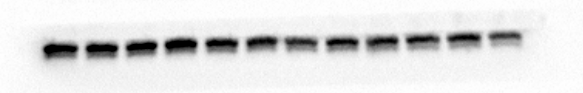


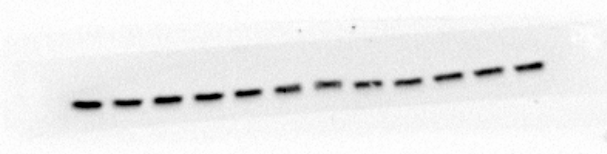


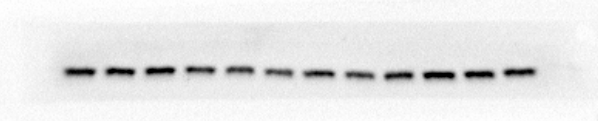


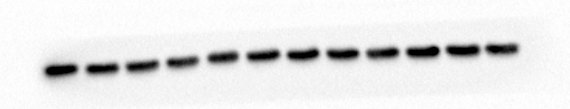


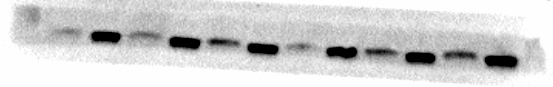


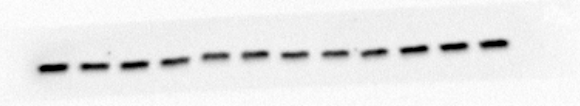


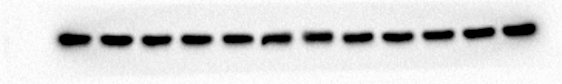


right


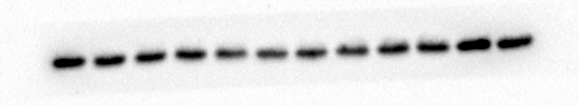


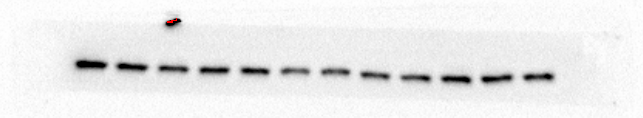


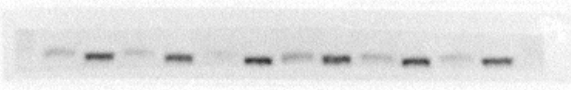


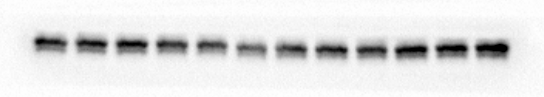


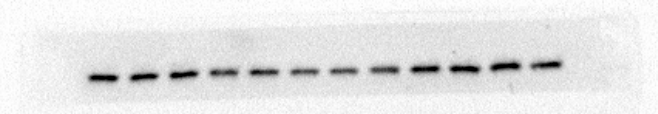


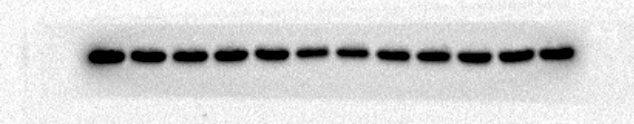


Figure4 F


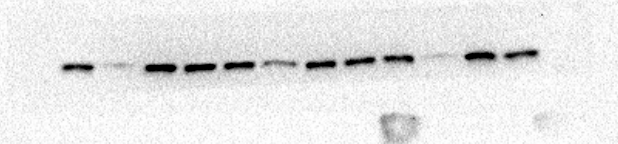


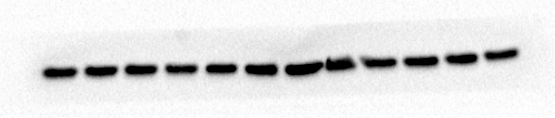


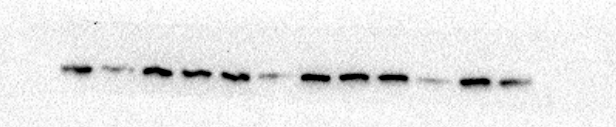


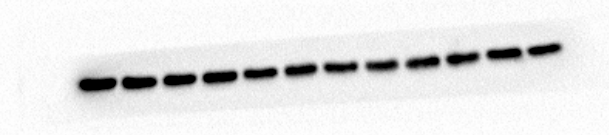


Figure4 H


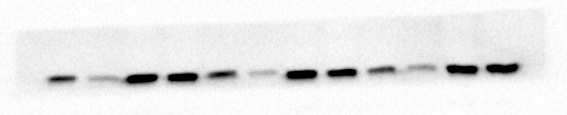


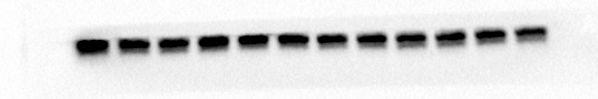


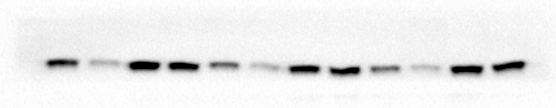


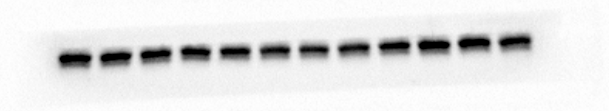


Figure4 N(cell group)


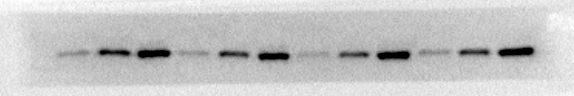


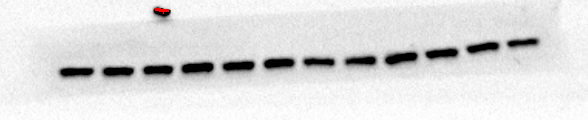


Figure4 N(human samples)


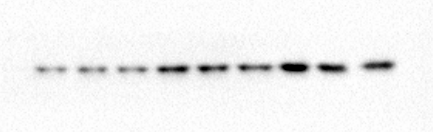


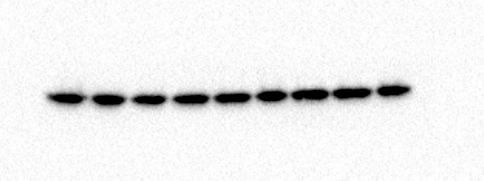


**Supplementary Figure4B (U251)**


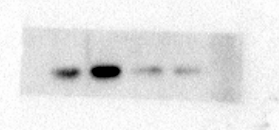


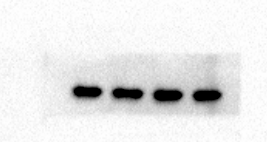


**Supplementary Figure4B (U87)**


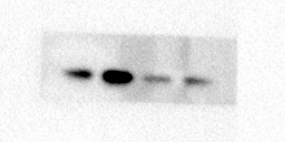


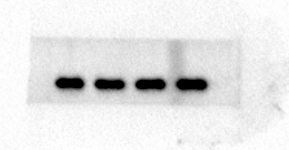


Figure5 B

U251


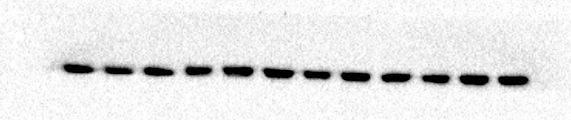


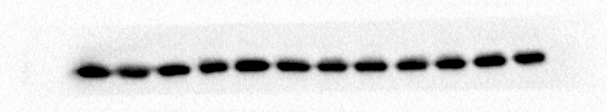


U87


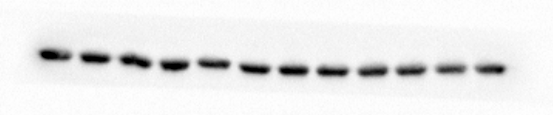


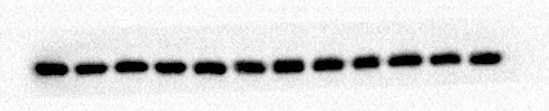


Figure5 C


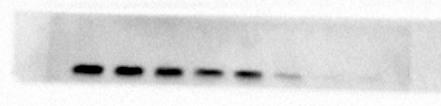


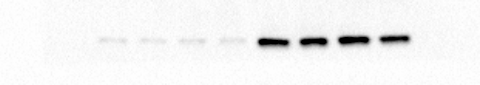


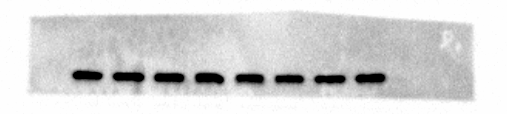


Figure5 D


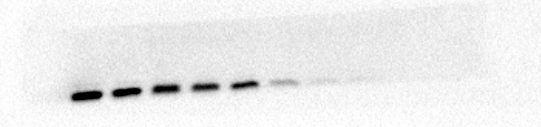


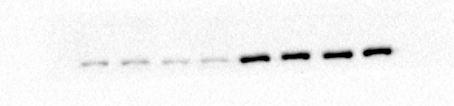


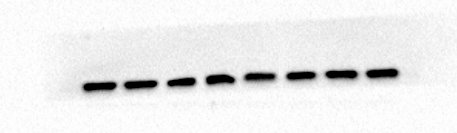


Figure5 E


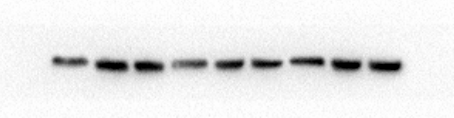


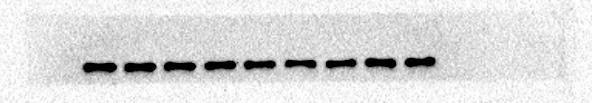


Figure5 F


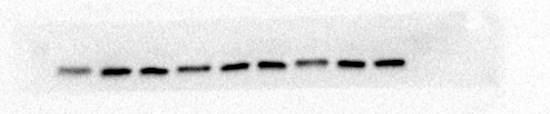


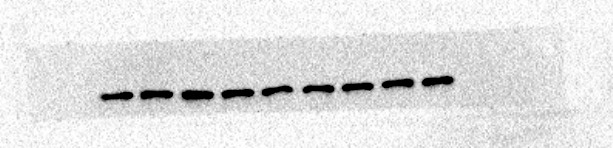


Figure5 G


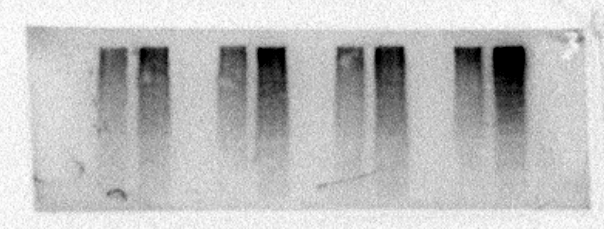


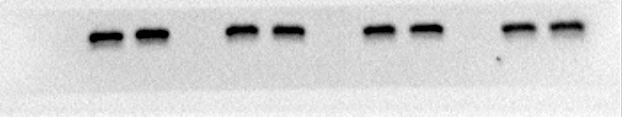


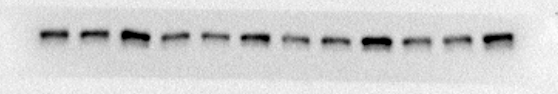


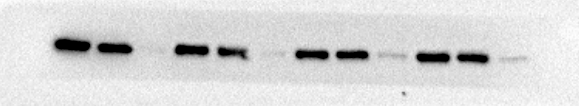


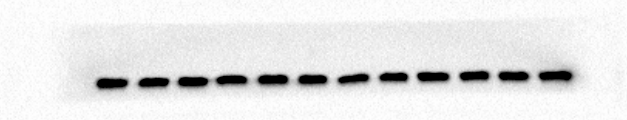


Figure5 H


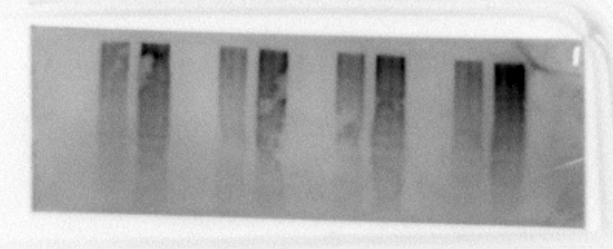


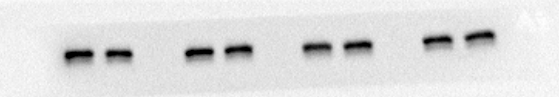


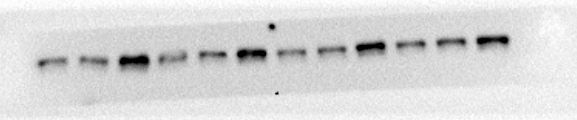


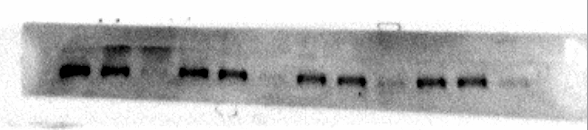


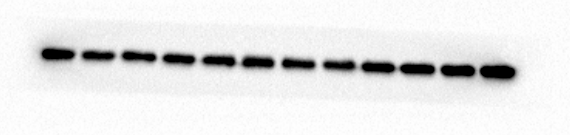


Figure5 I


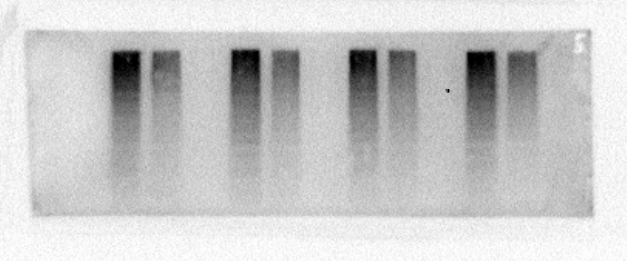


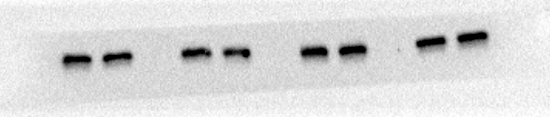


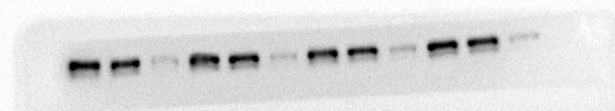


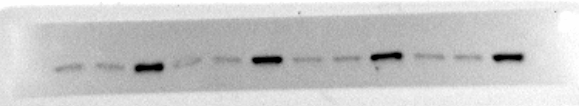


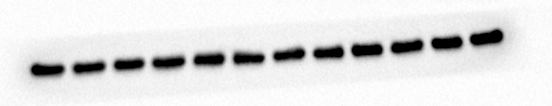


Figure5 J


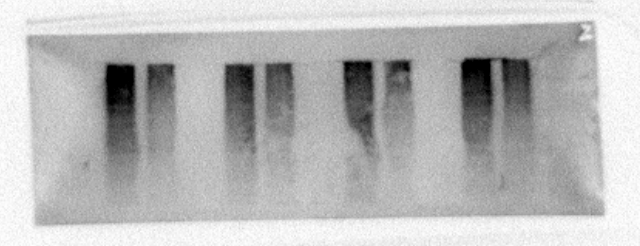


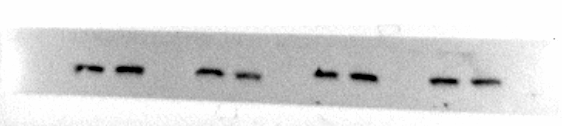


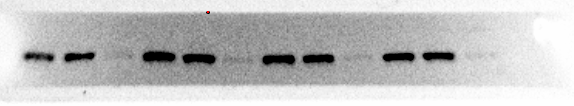


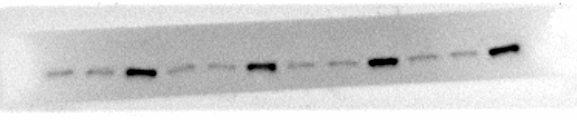


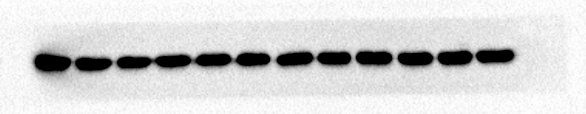

Supplement: Supplementary file 2 — Original Data File [file 41419_2023_6322_MOESM2_ESM.doc]
